# A selection and targeting framework of cortical locations for line‐scanning fMRI

**DOI:** 10.1002/hbm.26459

**Published:** 2023-08-22

**Authors:** Jurjen Heij, Luisa Raimondo, Jeroen C. W. Siero, Serge O. Dumoulin, Wietske van der Zwaag, Tomas Knapen

**Affiliations:** ^1^ Spinoza Centre for Neuroimaging Amsterdam Netherlands; ^2^ Department of Computational Cognitive Neuroscience and Neuroimaging Netherlands Institute for Neuroscience Amsterdam Netherlands; ^3^ Department of Experimental and Applied Psychology VU University Amsterdam Netherlands; ^4^ Department of Radiology University Medical Center Utrecht Utrecht the Netherlands; ^5^ Department of Experimental Psychology Utrecht University Utrecht Netherlands

**Keywords:** 7 T, BOLD fMRI, laminar, line‐scanning, pRF, ultra‐high field MRI

## Abstract

Depth‐resolved functional magnetic resonance imaging (fMRI) is an emerging field growing in popularity given the potential of separating signals from different computational processes in cerebral cortex. Conventional acquisition schemes suffer from low spatial and temporal resolutions. Line‐scanning methods allow depth‐resolved fMRI by sacrificing spatial coverage to sample blood oxygenated level‐dependent (BOLD) responses at ultra‐high temporal and spatial resolution. For neuroscience applications, it is critical to be able to place the line accurately to (1) sample the right neural population and (2) target that neural population with tailored stimuli or tasks. To this end, we devised a multi‐session framework where a target cortical location is selected based on anatomical and functional properties. The line is then positioned according to this information in a separate second session, and we tailor the experiment to focus on the target location. Anatomically, the precision of the line placement was confirmed by projecting a nominal representation of the acquired line back onto the surface. Functional estimates of neural selectivities in the line, as quantified by a visual population‐receptive field model, resembled the target selectivities well for most subjects. This functional precision was quantified in detail by estimating the distance between the visual field location of the targeted vertex and the location in visual cortex (V1) that most closely resembled the line‐scanning estimates; this distance was on average ~5.5 mm. Given the dimensions of the line, differences in acquisition, session, and stimulus design, this validates that line‐scanning can be used to probe local neural sensitivities across sessions. In summary, we present an accurate framework for line‐scanning MRI; we believe such a framework is required to harness the full potential of line‐scanning and maximize its utility. Furthermore, this approach bridges canonical fMRI experiments with electrophysiological experiments, which in turn allows novel avenues for studying human physiology non‐invasively.

## Graphical abstract

We present a selection and targeting framework of cortical locations for ultrahigh spatio‐temporal resolution line‐scanning fMRI. This approach bridges canonical fMRI experiments with electrophysiological experiments and allows novel avenues for studying human physiology non‐invasively.

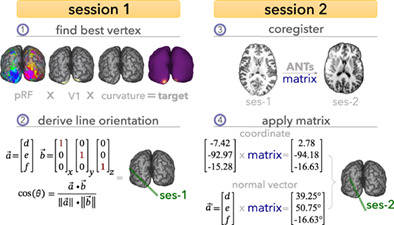

## 1 INTRODUCTION

The cerebral cortex comprises separate layers implicated in different processes and information flow. Imaging across cortical depth may reveal unique information about the direction of information flow, specifically whether processes are driven by ascending or descending signals (Felleman & Van Essen, 1991; Hubel & Wiesel, 1972; Rockland & Pandya, 1979). These signals are transmitted at time spans down to the millisecond range (Moro et al., 2010; Schroeder et al., 1998; Self et al., 2013), while typical functional magnetic resonance imaging (fMRI) acquisitions sample in the order of multiple seconds (Lindquist, 2008; Ogawa et al., 1993; Raimondo, Oliveira, et al., 2021). To properly detect temporal features of the hemodynamic signal propagation at the mesoscale (laminar) level, fast sampling rates are much preferred (Chen et al., 2021; Lewis et al., 2016; Petridou & Siero, 2019; Polimeni & Lewis, 2021; Silva & Koretsky, 2002). Additionally, the architecture of the human cortex requires high spatial resolution as well—with the cortex being 2 mm thick on average (Fischl & Dale, 2000), and visual cortex being among the thinnest cortical regions. To separate ascending from descending signals without relying on interpolation, submillimeter resolution is required (Dumoulin et al., 2018; Petridou & Siero, 2019). Ideally, we would go beyond what is presently considered “laminar” resolution at ultra‐high field (~0.8 mm; Dumoulin et al., 2018; Huber et al., 2015, 2021; Oliveira et al., 2022, 2023; Raimondo, Oliveira, et al., 2021). However, measurements at these resolutions are slow, and typically have a repetition time larger than 2 s (Raimondo, Oliveira, et al., 2021). To probe laminar properties *non‐invasively* more precisely in the *human* cortex, we can wield the power of line‐scanning (Choi et al., 2023; Raimondo, Knapen, et al., 2021; Yu et al., 2014). This acquisition technique allows for sampling rates down to ~100 ms and a spatial resolution of 250 μm in the line direction (frequency‐encoding direction), at the cost of spatial coverage (Raimondo, Knapen, et al., 2021; Raimondo, Priovoulos, et al., 2023).

In line‐scanning (Raimondo, Knapen, et al., 2021; Raimondo, Priovoulos, et al., 2023; Yu et al., 2014), a slice is excited and the signal outside the line of interest is suppressed through outer volume suppression (OVS) pulses (Pfeuffer et al., 2002). The phase‐encoding gradient in the direction perpendicular to the line is omitted, and the line signal is then acquired after every excitation pulse. This results in an acquisition with a spatial resolution of 250 μm in the laminar direction with a sampling rate of ~100 ms (Raimondo, Knapen, et al., 2021). In previous work, we showed BOLD responses along cortical depth in response to visual stimulation that were similar to 2D gradient‐echo echo planar imaging (GE‐EPI) acquisitions (Raimondo, Knapen, et al., 2021). This technique has striking similarities with laminar electrophysiological measurements used in rodent/non‐human primate research, where information is sampled from a single probe (Harris et al., 2016; Jun et al., 2017; Steinmetz et al., 2018). In that sense, we can perform fMRI experiments in the same way: if we know the target site of the line and its functional properties beforehand, we can tailor our experiments very specifically to the area being imaged.

Line placement is critical for line‐scanning. First, the line must be placed perpendicular to the cortex to avoid loss of spatial resolution in the cortical depth dimension. This ensures that only signals from a particular layer are sampled by a given voxel in the line (Balasubramanian et al., 2021, 2022). The first implementation of line‐scanning involved a rodent study (Yu et al., 2014). Due to the nature of the mouse cortex, planning the line perpendicular is a rather straightforward task. The cortex of humans has a much more intricate folding architecture (Van Essen et al., 2019), complicating (automatic) planning procedures. In earlier work (Raimondo, Heij, et al., 2023; Raimondo, Knapen, et al., 2021; Raimondo, Priovoulos, et al., 2023), this was done by manually placing the line as perpendicular as possible to the cortical surface while maintaining a coronal slice orientation. As the procedure is based purely on anatomy, it remains unclear whether the functional task will activate the imaged area and how the signal is sampled across depth. Second, line placement is critical to target the exact functional region the experimenters want to probe, for example, primary visual cortex (V1) or a specific region of V1 such as the cortical representation of the blind spot (Tong & Engel, 2001). Ultimately, line‐scanning can also be extended to clinical research, such as the lesion projection zone in V1 of macular degeneration patients where the balance between ascending or descending signals is disrupted (Baker et al., 2005; Masuda et al., 2008). The well‐known functional specialization of the brain and the variation of functional specialization domains between subjects, which can be in the order of ±1 cm relative to anatomical landmarks (Dumoulin et al., 2000), also demand accurate line placement on a per‐subject basis.

Therefore, the present work aims to (1) identify a specific location on the cortical surface, (2) place the line at this location perpendicular to the cortical sheet, and (3) tailor our experiment to that specific location on the cortical surface. We base the selection of the target and placement of the line on functional (visual field coverage, signal‐to‐noise) and structural (minimal curvature, avoiding veins) information across multiple sessions. Visual field coverage was quantified using the population‐receptive field (pRF) method (Dumoulin & Wandell, 2008), though the framework can be adapted for other purposes. We show that we are able to position the line on a specific coordinate obtained from a separate session. Anatomical variability such as session‐to‐session registration and subject motion were limited to <0.4 mm and ~0.6 mm, respectively. The pRF estimates obtained with line‐scanning were similar to the target estimates for most subjects. This was quantified by taking the distance from the target location and the location with most‐similar estimates compared to the line‐scanning estimates across primary visual cortex, showing an average displacement of ~5.5 mm, a displacement expected based on the width of the line.

## 2 MATERIALS AND METHODS

### 2.1 Participants

Six participants (ages 27–46 years, 2 females) participated in this study. All participants had normal or corrected‐to normal visual acuity. All participants were screened prior to the experiments to ensure MR compatibility and provided written informed consent as approved by the ethics committee of the VU University Amsterdam.

### 2.2 MRI acquisition and preprocessing

**FIGURE 1:**
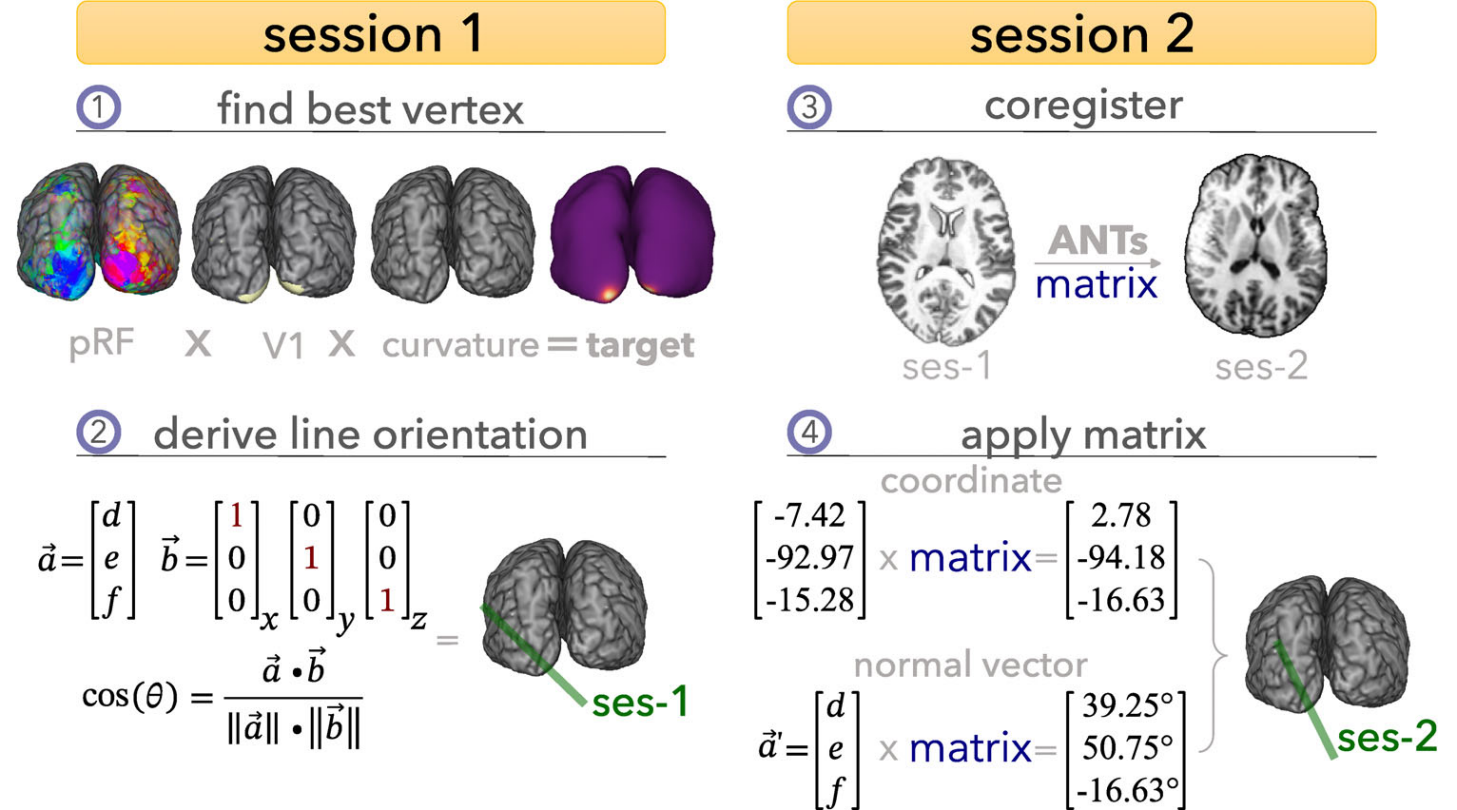
Schematic representation of the selection and targeting framework for line‐scanning. (1**)** In Session 1, we collect anatomical and functional data using standard sequences. Next, we reconstructed the cortical surface from the anatomical data and reconstructed the pRF properties from the functional data. Anatomical (curvature) and functional (pRF) properties were used to find a target vertex in primary visual cortex (V1). (2**)** The coordinate of the vertex was used as spatial reference; the normal vector was used to achieve perpendicularity to the cortex by calculating the angle between the normal vector and each cardinal axis (x, y, z). (3**)** Session 2 started with a brief, low‐resolution anatomical scan, which was exported from the scanner, and registered to the detailed anatomy from Session 1. (4) The resulting transformation was applied to the coordinate and normal vector of the target vertex resulting in the coordinates and orientation for the line.

The workflow includes two separate scan sessions typically acquired on different days (Figure 1). The first session is dedicated to the acquisition of anatomical information and whole‐brain pRF estimation. In the second session, we perform our functional line‐scanning experiment, targeting a specific location on the cortical surface. Details of Session 1 are described in Section 2.2.1. Section 2.2.2 describes the method of deriving the target vertex and angulation of the line, and Section 2.2.3 describes the line‐scanning acquisition, experiment, and analysis.

#### 2.2.1 Session 1—High‐resolution anatomical scans and pRF mapping

##### High‐resolution anatomy and whole‐brain BOLD fMRI acquisition

T1‐weighted (T1w) and T2‐weighted structural MRI data were acquired using a Philips Achieva 7T scanner with a 32‐channel Nova Medical head coil, at a resolution of 0.7 mm isotropic (*T1w*: FOV = 220 × 220 × 200 mm^3^, matrix = 352 × 352 × 263, TR/TE = 6.2 ms/3 ms, FA_1_/FA_2_ = 5°/7°, TR_MP2RAGE_/TI_1_/TI_2_ = 5500 ms/800 ms/2700 ms, duration = 9 min 45 s; *T2w*: FOV = 245 × 245 × 184 mm^3^, matrix = 352 × 349 × 263, TR/TE = 3000/390 ms, TSE‐factor = 182, duration = 7 min). Functional MRI data were acquired with a 1.7 mm isotropic T2*‐weighted gradient‐echo (GE‐) 2D‐EPI sequence with 57 slices and 225 volumes (FOV = 216 × 216 mm^2^, matrix = 128 × 125, TR/TE = 1500 ms/22 ms, FA = 53°), with a duration of 330 s. Six dummy scans (9 s) were discarded to avoid start‐up magnetization transients. Foam padding was used to minimize head movement. At the end of each functional run, a top‐up scan with opposite phase‐encoding direction was recorded, in order to perform susceptibility distortion correction (Andersson et al., 2003).

##### pRF experiment

We used a bar‐shaped stimulus with a checkerboard pattern vignetted by a circular aperture (Aqil et al., 2021; Dumoulin & Wandell, 2008). Four bar orientations (0°, 45°, 90°, and 135°) and two different motion directions were used, giving a total of eight different bar traversal configurations. The width of the bar subtended 1.25°. A period of 15 s of mean luminance was presented every two bar passes. Participants' engagement was ensured by presenting a small fixation dot in the middle of the stimulus that changed color (red–green) at a semi‐random interval. Participants were instructed to report this change of color via a button press.

##### Anatomical workflow

T1w and T1 map images (Marques et al., 2010) were estimated using pymp2rage (de Hollander, 2018), and voxels containing only noise were removed using a brain mask derived from the second inversion image (INV2) using SPM12. The resulting T1w anatomical image was processed following a pipeline designed to optimize laminar accuracy (de Hollander et al., 2021; Figure S1).

First, a spatial‐adaptive Non‐Local Means (*SANLM*‐) filter implemented in CAT12 was applied to the T1w‐images to filter noise while maintaining edges (Manjón et al., 2010). The denoised image was segmented into cerebrospinal fluid (CSF), white matter (WM) and gray matter (GM) using CAT12 and corrected for intensity non‐uniformity with *N4BiasFieldCorrection* (Tustison et al., 2010), distributed with ANTs 2.3.3. A mask representing the sagittal sinus was created using the T1w/T2w (if present) ratio and further refined by hand. The voxels in the mask were set to zero in the denoised T1w image to limit the necessity for manual intervention after surface reconstruction. The final masked image was then used as input for the structural preprocessing module of fMRIprep (Esteban et al., 2019), where the image was skull‐stripped with a Nipype implementation of the *antsBrainExtraction.sh* workflow (Avants et al., 2008), using OASIS30ANTs as target template. Brain tissue segmentation of CSF, WM, and GM was performed on the brain‐extracted T1w using FSL's *FAST* (Zhang et al., 2001). FreeSurfer 7.2 *recon‐all* (Dale et al., 1999) was used to obtain native cortical surface reconstructions for each participant.

After surface reconstruction, segmentations derived from *FreeSurfer* (Dale et al., 1999), *CAT12*, *FAST* (Zhang et al., 2001), Nighres' *MGDM* (Bazin et al., 2014; Bogovic et al., 2013), and manual edits were averaged and used as input for Nighres' *CRUISE* algorithm (Han et al., 2004). Mislabeled voxels were manually annotated in ITK‐Snap (Yushkevich et al., 2006) and added to FreeSurfer's *brainmask.mgz*, after which the entire preprocessing pipeline was run again. This process was repeated until surface reconstruction was satisfactory (Figure S1).

##### Functional workflow

After surface reconstruction, whole‐brain functional MRI data were preprocessed using fMRIPrep (see Supporting Information Methods for boilerplate). Confound regressors were removed from the preprocessed BOLD time courses using pybest. We then converted the data to %change using the mean of the empty‐screen periods in the pRF experiment. Denoised, %changed BOLD time courses were averaged across runs and used to estimate pRF parameters by means of a Gaussian pRF (Dumoulin & Wandell, 2008; Figure S2), implemented in prfpy. This model contains of three spatial parameters, $x_{0}$, $y_{0}$, and σ, where ($x_{0}$, $y_{0}$) is the center and σ is the Gaussian spread (standard deviation). For a given pRF model, a prediction of the pRF response is obtained by taking the dot product between pRF and stimulus at each timepoint and convolving this with the hemodynamic response function. The optimal pRF parameters were found by minimizing the residual sum of squares (SoS) with *trust‐contr* optimization (Figure S2).

#### 2.2.2 Vertex selection

Vertex selection was implemented making use of surface processing procedures from pycortex (Gao et al., 2015). From the pRF‐parameters obtained, we calculated the eccentricity of each vertex and polar angle. As criteria, we used an eccentricity <3° of visual angle, with high variance explained (depending on subject; range 0.35–0.7) and searched within V1 (as per the *V1_exvivo.thresh* label from FreeSurfer) for the vertex with minimal curvature. This vertex is associated with an RAS coordinate in FreeSurfer (*TKR*) space. This coordinate was then transformed to scanner coordinates using Option 5 of https://surfer.nmr.mgh.harvard.edu/fswiki/CoordinateSystems. To position the line perpendicular to the selected patch of cortex, we aimed to place the line along the normal vector of the vertex (see Section 2.2.3, “Line‐planning procedure” for more details) at mid‐gray‐matter depth.

#### 2.2.3 Session 2—Line‐scanning

##### Line‐scanning fMRI acquisition

The line‐scanning functional acquisition used a modified multi‐echo 2D gradient‐echo sequence where the phase‐encoding gradients are removed and two OVS bands are used to suppress signals outside the line (Raimondo, Heij, et al., 2023; Raimondo, Knapen, et al., 2021). With this sequence, 94.3 ± 1.3% of undesired signal outside the region of interest is suppressed (Raimondo, Knapen, et al., 2021, Raimondo, Priovoulos, et al., 2023). A gap of 4 mm between the two OVS bands was used, resulting in a nominal resolution for the line of 4 × 2.5 × 0.25 mm^3^; thus, 0.25 mm in the laminar direction. Other parameters were: TR/TE_1–5_ = 105 ms/6 ms, 14 ms, 22 ms, 30 ms, 38 ms, readout bandwidth = 131.4 Hz/pixel, FA = 16° (Raimondo, Priovoulos, et al., 2023). Data were acquired using two custom‐built high‐density 16‐channel surface coils arrays (total 32 channels) for signal reception (Petridou et al., 2013; Priovoulos et al., 2021) and the NOVA coil for transmission (Nova Medical, Wilmington, MA). The gradient coil has a maximum amplitude of 40 mT/m and a 200 T/m/s maximum slew rate.

For registration, a 4‐min whole‐brain T1w scan was acquired using the two‐channel transmit coil to receive (Nova Medical, Wilmington, MA), at a resolution of 1.5 mm isotropic (FOV = 245 × 245 × 184 mm^3^, matrix = 164 × 163 × 184, TR/TE = 6.2 ms/3 ms, FA_1_/FA_2_ = 5°/7°, TR_MP2RAGE_/TI_1_/TI_2_ = 5500 ms/800 ms/2700 ms). A partial field‐of‐view MP2RAGE scan (FOV = 245 × 245 × 184 mm^3^, matrix = 164 × 163 × 184, resolution = 1.5 × 1.5 × 2.0 mm^3^, TR/TE = 6.2 ms/1.97 ms, FA = 6°) was acquired with the angulation and location of the line for anatomical reference. Two short additional scans accompanied the line‐scanning acquisition: for the nominal line representation, a slice image *with* phase‐encoding, but *without* OVS bands was acquired. For line coil sensitivity maps used when reconstructing line‐scanning data, a slice image *with* phase encoding and *with* OVS bands was acquired.

##### Line‐planning procedure

Immediately upon completion of the low‐resolution anatomical scan, we exported the image and registered it to the first session using rigid‐body registration with *antsRegistration* (ANTs v2.3.1). The resulting transformation matrix was applied to the coordinate (using *antsApplyTransformsToPoints*), allowing us to obtain the normal vector following the procedure detailed in Section 2.2.2. The angle between the normal vector and each of the three axes was calculated using the rule of cosines (Figure 1). To translate these angles to the magnet (imaging gradients) coordinate frame of reference, we re‐calculated the angles relative to a coronal slice. If the angle with the left–right axis was larger than 45°, we re‐calculated the angles relative to a sagittal slice. The final set of orientations/translations was then entered in the MR‐console for subsequent line‐scanning at the targeted location.

##### pRF experiment

**FIGURE 2:**
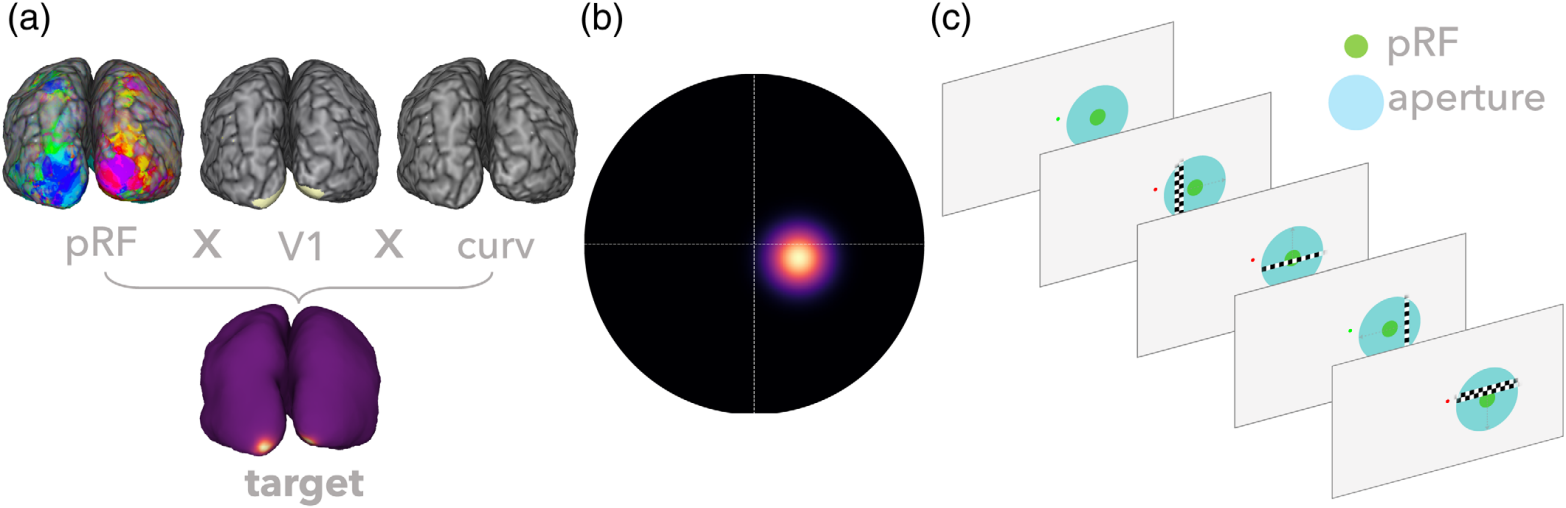
Overview of the experimental setup. From structural and functional properties, a target vertex was derived (a). This target vertex represents a particular portion of the visual field (b). We then tuned the experimental design to target that specific location in visual space (c). pRF, population receptive field.

During the line‐scanning session, we performed a pRF‐experiment targeting the visual location of the target vertex' pRF (Figure 2). The visual stimuli were generated using the Python Psychopy package (Peirce, 2007) wrapped in exptools2. Stimuli were displayed on an MRI‐compatible screen located outside the bore (Cambridge Research Systems 32" LCD widescreen, 1920 × 1080 resolution, 120 Hz refresh rate), viewed by the participants through front‐silvered mirrors. Two bar orientations (0° and 90°), two motion directions, and two bar thicknesses (0.625° and 1.25°) were used, giving a total of eight different bar configurations (duration of ~5 min); two iterations of an identical stimulus movement sequence were performed per run, resulting in an acquisition time of ~9.5 minutes. Participants' engagement was ensured using the same task as described in Section 2.2.1, pRF experiment.

##### Preprocessing and analysis

The reconstruction of the line‐scanning data was performed offline using MATLAB Gyrotools. We combined the multi‐channel coil data with a temporal signal‐to‐noise ratio (tSNR) and coil sensitivity‐weighted SoS weighted scheme per echo (Raimondo, Knapen, et al., 2021, Raimondo, Priovoulos, et al., 2023). Prior to channel combination, we applied a NORDIC‐inspired denoising step (Vizioli et al., 2021). Briefly, raw k‐space data of each channel and echo was subjected to singular value decomposition separately. The thresholding of the diagonal matrix with the eigenvalues was evaluated at the elbow of the scree plot of the eigenvalues versus components (Raimondo, Priovoulos, et al., 2023). Multi‐echo data were then combined with a sum of squares operation (Raimondo, Priovoulos, et al., 2023).

Drifts were removed from the data using a discrete cosine transform (DCT) filter (<0.01 Hz) and physiological noise was removed using a custom implementation of *aCompCor* (Behzadi et al., 2007), tailored to line‐scanning data (Figure S3): tissue segmentations from Nighres' *CRUISE* algorithm (Han et al., 2004) from the Session 1 anatomical scan were transformed to the individual slices of each run using *antsApplyTransforms* with *MultiLabel* interpolation. WM and CSF voxels were selected by multiplying the slice with the nominal line image. Time courses from these voxels were extracted and used as input for principal component analysis (PCA). To avoid task‐related frequencies being regressed out, resulting time courses from the PCA were high‐pass filtered slightly below the respiratory frequency (~0.18 Hz). These high‐pass filtered time courses were used as nuisance regressors to clean the data from respiration/cardiac frequencies (Behzadi et al., 2007; Figure S3). The cleaned time courses were then converted to %change.

Because this experiment reflects relatively slow changes in visual processes, the temporal resolution was not fully exploited. Hence, for further noise reduction, we low‐pass filtered the data with a Savitsky–Golay filter (Savitzky & Golay, 1964) with a window length of 11 samples and a polynomial order of 3. To further boost SNR, we averaged the two iterations of the experimental stimulus sequence from each run.

## 3 RESULTS

### 3.1 Anatomical measures confirm accurate line‐planning

**FIGURE 3:**
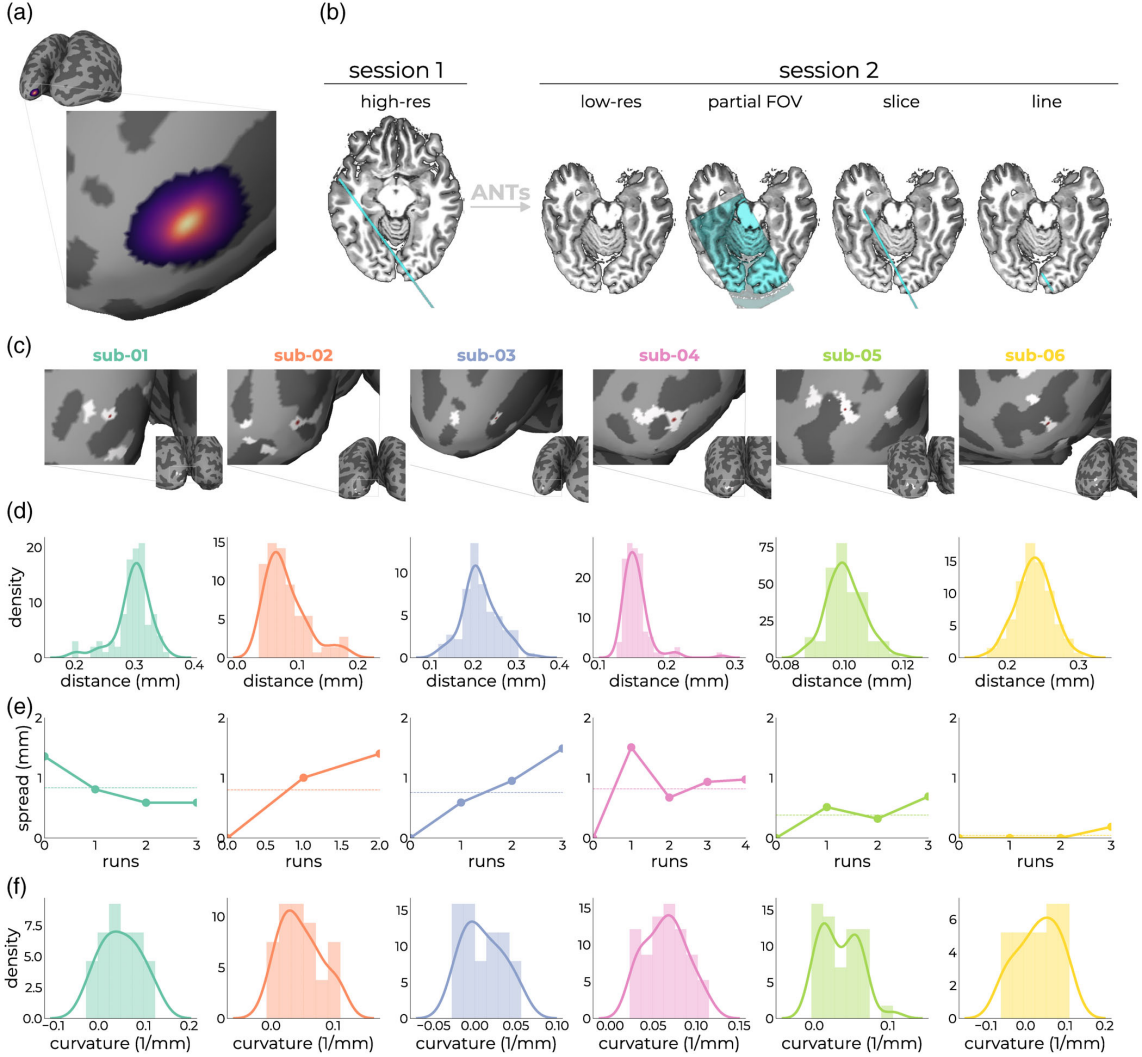
Assessment of line‐placement accuracy using anatomical measures. The registration cascade from target vertex (a) to line‐scanning acquisition (b; outer right panel) is known after registering the anatomical image from Session 1 (high‐res) to the anatomical image from Session 2 (low‐res). Within Session 2, we acquired a partial field‐of‐view image (partial FOV), as well as the anatomical slice without OVS pulses (slice). From this slice, we created an image representing the nominal line (line). For each subject, we projected this nominal line image back to the surface from which the target vertex originated, showing sufficient overlap between the target vertex (red dot) and nominal line image (white patches) (c). The patches represent the location at which the nominal line image intersects with GM and looks scattered due to unfolding of the cortex. (d) Variation in registration outcomes after registration anatomies from Sessions 1 and 2, a hundred times for each subject. (e) Effect of subject motion by means of manual alignment of the single slice images on positional stability of the target coordinate. (f) Curvature distributions within the gray patch intersecting with the target location of (c). The curvature is measured as 1/r, where *r* is the radius of an inscribed circle. Since mean curvature is the average of the two principal curvatures, it has the units of 1/mm (https://surfer.nmr.mgh.harvard.edu/fswiki/MeanCurvature).

First, we used anatomical information to assess planning accuracy. We created a binarized mask representing the nominal line by taking the middle 16 voxels (indicative of the nominal 4 mm gap between saturation pulses) in the phase encoding direction along the frequency encoding direction. Of note, due to the imperfect nature of these saturation slabs, there are contaminating signals coming from outside the region‐of‐interest (Raimondo, Knapen, et al., 2021; Raimondo, Priovoulos, et al., 2023). Because the full registration cascade from target vertex to the line through the low‐resolution image, partial FOV image, and single slice is known, we can project the nominal line image back onto the surface (Figure 3a,b). This showed that for all subjects, the line was indeed placed on the target site as per the overlap of the line (white) and target vertex (red spot) (Figure 3c).

One potential source of variation is the registration accuracy between anatomical images from the first and second session. For each subject, we performed the registration 100 times, applied the resulting matrices to the target coordinate, and calculated the Euclidean distance to the original coordinate targeted in the second session. Thus, for each subject, we obtained a distribution representing registration variation. This procedure showed that registration was highly stable (<0.4 mm) for all subjects (Figure 3d). An additional source of variation is subject motion. Because of the limited field‐of‐view single slices offer, it is challenging to estimate this accurately. Nevertheless, we manually aligned slices of each run to the first slice right after the first anatomical scan. This procedure assumes motion is limited after initial registration of the first and second session and the acquisition of this slice. We applied each run‐to‐run transformation matrix to the targeted coordinate with *antsApplyTransformsToPoints* to obtain the coordinate in each run‐specific slice. We then evaluated the Euclidean distance between these coordinates and the initial target coordinate. This showed that motion induced an average displacement of the target coordinate of ~0.6 mm across runs (Figure 3e).

In the current line‐planning implementation, the target coordinate was selected by minimizing the curvature in a patch of cortex that survived the initial pRF‐criteria. For each subject, we estimated the distribution of curvature values present in the line (Figure 3f). This showed that on average, the curvature was predominantly flat (0.04 ± 0.003 1/mm). For some subjects, the initial criteria (e.g., variance explained and/or visual field position) moved the area within which to optimize for curvature towards areas with more curvature. This necessarily results in a loss of spatial specificity within the line; signals coming from non‐GM were mixed into the line. From manual segmentations, we estimated that the voxels covering the cortical ribbon around the target region consisted of 88.34 ± 1.07% GM, 8.22 ± 2.77% WM, and 3.44 ± 3.18% CSF (Figure S5). These estimations are likely conservative given the imperfect OVS bands.

### 3.2 Functional measures confirm accurate line‐planning

**FIGURE 4:**
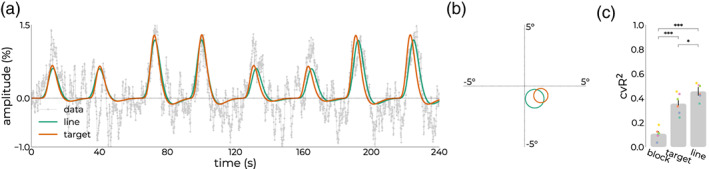
Predicted responses (a). In orange, the predicted time course obtained by passing the design matrix of the pRF paradigm during the line‐scanning experiment through the estimates of the target vertex. In green, the prediction given by the model estimates after fitting the actual line‐scanning data. (b) The corresponding predicted pRFs in visual space for the target vertex estimates (orange) and fitted estimates (green). (c) Cross‐validated variance explained (cvR^2^) across subjects for a design that is spatially invariant (block), the line‐scanning design given the target vertex estimates (target), and line‐scanning estimates (line). *p < .05, ***p < .001. pRF, population receptive field.

We next assessed line localization based on functional properties. First, we predicted the signal of the target vertex (Figure 4a, orange curve) in response to the stimulus design during the line‐scanning experiment. To deal with potential differences in BOLD amplitudes across sessions and sequences, we performed an additional GLM between the prediction from the target vertex' pRF and line‐scanning data. This resulted in a strong overlap between this prediction and the estimated prediction from fitting the line‐scanning data (Figure 4a, green curve). Despite this marked overlap, we observed slight variations in pRF size and location (Figure 4b). To estimate the out‐of‐experiment variance explained (*r* ^2^), we compared the *r* ^2^ from the target vertex prediction and the estimates from the line‐scanning fits, and benchmarked this against the null‐model that predicts a signal time course assuming a response that is not spatially selective, that is, a block design reflecting activation whenever the stimulus is on the screen, regardless of position (Figure 4c). The variance explained from the null‐model (*block*) was significantly lower than the *target* prediction (*t*_10_ = −6.19, *p* < .001, Cohen's *D* = −3.57) and *line* prediction (*t*_10_ = −10.67, *p* < .001, Cohen's *D* = −6.16), whereas the difference between *target* and *line* prediction was significant but only for a one‐sided effect (*t*_10_ = 2.30, *p* < .044, Cohen's *D* = 1.33). Overall, pRF estimates between target vertex and line‐scanning estimates were very similar (Figure 4a).

**FIGURE 5:**
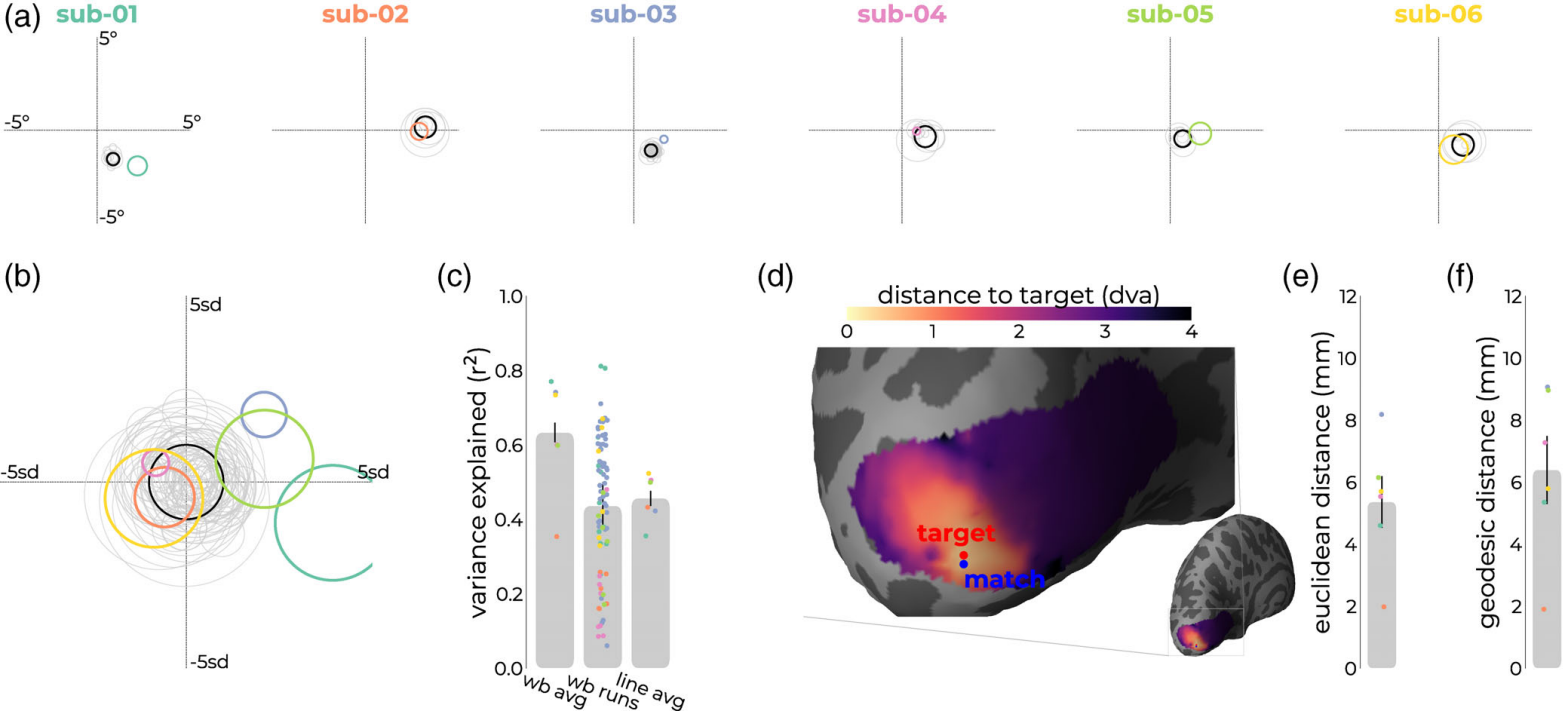
Overview of functional confirmation metrics. (a) For all subjects, the target vertex averaged across runs (black) and individual runs (gray) from the first session, and the estimated pRF of the second (line‐scanning) averaged across runs, within‐run iterations, and gray matter voxels around target location. (b) Shows a normalized version of (a), in which line‐scanning pRFs shifted relative to the target vertex' pRF, and their sizes are divided by the target vertex' pRF size (standard devation [sd]). (c) Variance explained for the target vertex averaged across runs (wb avg), individual runs (wb runs), and line‐scanning data (line avg). (d) For each vertex in V1, we assessed the distance from the vertices' pRF to the line‐scanning pRF in visual space. pRFs closer to the line are represented by lighter colors, while pRFs farther away show up in darker colors. We then assessed where in V1 the pRF from the whole‐brain data (blue) matched best with the target vertex (red) in terms of Euclidean (e) and geodesic (f) distance. pRF, population receptive field.

pRF estimates of location obtained with line‐scanning were similar to the estimates of the target (Figure 5a,b) for both position and pRF size. Variance explained for line‐scanning averaged over runs was comparable to that of single run whole‐brain acquisitions (Figure 5c). To assess the similarity between target pRF and line‐scanning pRF, we obtained the distance between the centers of the target pRF (*target*) and all vertices in V1 and picked out the vertex that was closest to the line‐scanning pRF (*match*). Reflecting V1's retinotopic organization, we observed that the further away from the target we move along the cortex, the more divergent the pRFs become (Figure 5d). We then calculated the distance between this matching and target vertex on the surface (Figure 5e,f; Figure S4). Given that the line is defined by the gap between saturation slabs (~4 mm) and the thickness of the slice (2.5 mm), the distance of the matching pRF to the target pRF should ideally be limited to the surface area of this rectangular surface patch. For most subjects, we found that the pRFs indeed fell within these dimensions (1.97–8.18 mm, Figure 5e).

**FIGURE 6:**
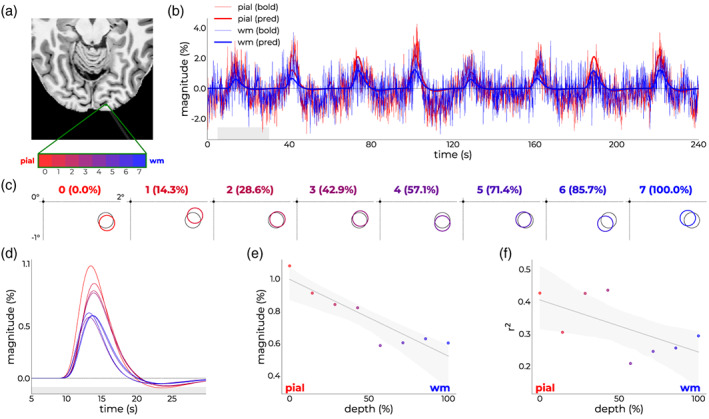
Single‐subject depth‐dependent outcomes. (a) Axial slice from T_1_‐image with the imaged line in white shading. Independent voxels covering the cortical ribbon are annotated in green and assigned a color ranging from red (superficial) to blue (deep). (b) Raw time courses (thin lines) and model predictions (thick lines) for a superficial (red) and deep (blue) voxel. (c) Position estimates in the bottom right quadrant of the visual field from voxels covering the cortical ribbon in color that scales from red (0% depth—superficial) to blue (100% depth—deep). Note that all time courses were modeled independently. (d) Response profile of the first bar pass across depth (same color coding as c); inset shows the magnitude. (e) Magnitude and (f) variance explained as a function of depth.

For one of these subjects, we show a more detailed profile of depth‐dependent measures by modeling all voxels covering the ribbon *independently* (Figure 6a). For this subject, the time courses of superficial and deep layers exhibited the classical pattern (Figure 6b), where the magnitude of the superficial time courses was almost double the magnitude of the deeper time course. The position estimates were stable across cortical depth (Figure 6c), with a slight non‐systematic scatter around the average—similar to early electrophysiological (Hubel & Wiesel, 1974) and fMRI (Fracasso et al., 2018) results. The response magnitude (Figure 6d,e) and variance explained (Figure 6f) scaled with cortical depth, an effect often reported in fMRI literature due to ascending draining veins (de Hollander et al., 2021; Fracasso et al., 2018; Koopmans et al., 2011; Lawrence et al., 2019; Polimeni et al., 2010; Self et al., 2019; Siero et al., 2011; van der Zwaag et al., 2018; van Dijk et al., 2020).

## 4 DISCUSSION

Here, we present a framework for the neuroscientific implementation of high spatiotemporal resolution line‐scanning fMRI. This was done by targeting a specific patch of cortex and tailoring the visual experiment to the properties of this patch. In this work, we applied the method in the context of pRFs; based on whole‐brain pRF mapping, a target vertex was selected. The pRF‐mapping experiment for the line‐scanning was focused on its representation in visual space. The line was placed on the associated coordinate and along the normal vector, that is, perpendicular to the cortex, which limits mixing of signals coming from different cortical depths. The accuracy of this framework was quantified based on (1) anatomical and (2) functional measures.

To anatomically confirm the location, we projected the image representing the nominal line back to the surface from which the target vertex originated. This showed good overlap between the line and the target vertex, suggesting we indeed hit the intended coordinate. Such an approach is subject to multiple sources of variation, such as registration accuracy and subject motion. We found the registration between Sessions 1 and 2 to be accurate within <0.4 mm, which could be attributed to the selection of transformation and regularization models within ANTs (Avants et al., 2008, 2011). Another source of anatomical variability is subject motion; because of the limited field‐of‐view of line‐scanning, it is particularly prone to this type of variability as spatial references are severely reduced (Balasubramanian et al., 2021; Raimondo, Knapen, et al., 2021; Raimondo, Priovoulos, et al., 2023). To limit problems regarding motion, the subject pool mostly consisted of subjects who had extensive experience with MRI (Balasubramanian et al., 2021). The usual foam padding to fixate the head was used as well. Regardless, we assessed motion throughout the session by using manual alignments of the single slices with phase‐encoding direction and without OVS bands, showing the target coordinate shifted about 0.6 mm throughout the session.

The functional accuracy was assessed by estimating the distance between the target vertex and the vertex whose whole‐brain pRF estimates matched the obtained line‐scanning estimates best. In a sense, this quantified the extent to which line‐scanning can produce similar estimates compared to whole‐brain data. On average, the best‐matching vertex was 5.5 mm away from the target coordinate. Given the dimensions of the line thickness (i.e., not its cortical depth resolution) and (imperfect) gap between saturation slabs, this result was well within the expected bounds.

Though we scanned at a spatiotemporal resolution of 100 ms and 250 μm, the effective resolution will be lower due to several factors impacting spatial and temporal resolution (Chen et al., 2019). First, the amount of curvature in the line affects the effective spatial resolution (Leprince et al., 2015; Shamir et al., 2019; Trampel et al., 2019). Our approach optimizes for minimal curvature after including structural and functional properties. The corollary of such an approach is that curvature is not always exactly flat, causing signal coming from the WM and CSF being mixed into the deeper and superficial layers, respectively. In our dataset, CSF and WM contributions were 11%. This comes, however, with the advantage that the method can be applied to any subject, including patients. As previously described, subject motion also poses challenges to line‐scanning in both the spatial and temporal domain (Godenschweger et al., 2016; Zaitsev et al., 2015). In previous work, we have shown the effect of prospective motion correction (PMC) using different flavors of navigators (Raimondo, Priovoulos, et al., 2023), highlighting the use of PMC in MRI‐naïve subjects using *block* paradigms. In such paradigms, the T_1_‐decay induced by the navigators can be clearly observed and separated from task‐signals. The full potential of line‐scanning lies in its ability to sample extremely fast. Therefore, event‐related designs are desired to evaluate response shapes to different stimuli (Dale, 1999; Mumford et al., 2015). In such scenarios, it will be challenging to separate task‐related signals from navigator‐induced signals. Another possibility for PMC involves the use of external hardware (Godenschweger et al., 2016; Maclaren et al., 2012; Schulz et al., 2012; Stucht et al., 2015). Finally, because of the high sampling rate (~0.1 Hz), cardiac and respiratory frequencies are resolved (Chen et al., 2019). This allows us to accurately characterize physiological noise due to cardiac and breathing fluctuations and target these frequencies for removal using aCompCor (Behzadi et al., 2007). In our experience, this method is more effective (and stable) for line‐scanning data than using regressors from pulse‐oximeter/respiration belt recordings.

Several factors can contribute to differences in pRF estimates (Dumoulin & Knapen, 2018; Dumoulin & Wandell, 2008). First, we changed the aperture size from full‐field (in Session 1) to localized around the pRF of the target. pRF estimates have been shown to shift away from their preferred location when reducing the stimulus extent (Kay et al., 2013; Prabhakaran et al., 2020). Second—and related to the previous point—attentional modulation might occur by changing the visual extent and aperture location; changing the aperture might induce an involuntary shift of attention towards the border of the stimulus (exogenous) that could result in pRF variations (Alvarez et al., 2015; Klein et al., 2018; Prabhakaran et al., 2020; van Es et al., 2019; Womelsdorf et al., 2006, 2008). Third, the pRFs were estimated from different sequences and across different days. Though pRF estimates are typically robust under identical conditions, inducing scanner‐related sources of variation may contribute (Alvarez et al., 2015; Lage‐Castellanos et al., 2020; Senden et al., 2014; van Dijk et al., 2016). The spatial resolution of line‐scanning in laminar direction^1^ is much higher compared to the whole‐brain acquisition, which has two side effects affecting pRF estimates; (1) it alters the neuronal population that is contributing to the response to a given stimulus (Dumoulin & Knapen, 2018); and (2) at these resolutions, the signal is more dominated by thermal noise (Bianciardi et al., 2009; Raimondo, Priovoulos, et al., 2023; Triantafyllou et al., 2005). As pRF estimation is a non‐convex problem, the cost function presents numerous local minima that compromise the convergence of optimization algorithms (Benson et al., 2018; Lage‐Castellanos et al., 2020). This problem is particularly relevant in noisy voxels, where one of the local minima can be mistaken for the global minimum, and thus result in compromised estimates of the pRF size in particular (Benson et al., 2018; Lage‐Castellanos et al., 2020). We tried to mitigate this effect by temporally smoothing our data, which increase variance explained measure and stabilizes model estimation, yet may inflate pRF size estimates (Morgan & Schwarzkopf, 2020). In future applications of line‐scanning, we will also exploit the temporal aspect. Given all these factors, some spread in pRF estimates was expected and the estimates are quite close.

In all, we have demonstrated the ability to target a specific location in cortex allowing the functional properties of this location to be probed. The implementation described in this work included two sessions. The advantage of such an approach is that subjects (including patients) can be selected, and lines planned, based on existing datasets and arbitrary functional properties probed in these experiments. Theoretically, however, it is possible to conduct such experiments within a single session. However, certain steps along the pipeline—including surface reconstruction and pRF modeling—take a significant amount of time. Several concessions could be made, typically at the cost of accuracy. For instance, several software packages such as BrainVoyager (Goebel et al., 2006), CAT12 (https://neuro-jena.github.io/cat/), or FastSurfer (Henschel et al., 2020), are able to create surfaces quickly (15–60 mins). These packages might encode coordinates differently, so the exercise becomes translating those coordinate systems to the coordinate system of the scanner. Likewise, approaches such as DeepRF (Thielen et al., 2019) or fast, real‐time pRF mapping (Bhat et al., 2021) can reconstruct pRFs based near real‐time. These approaches demand significant resources from the software as well as skills from the experimenter. Currently, the most robust implementation requires two sessions.

Though we used line‐scanning in the context of pRFs, our approach is general and can be extended to other modalities. For instance, in the context of (cerebrovascular or neurodegenerative) disease; if a lesion map is available, the line could be placed such that it targets exactly this lesion. Alternatively, if a given location within the vasculature is compromised, a line could be placed right at the problematic area to probe microvascular flow patterns (Angleys et al., 2015; Gutiérrez‐Jiménez et al., 2018; Østergaard et al., 2013; Rasmussen et al., 2015; Zwanenburg & van Osch, 2017). The line could also be placed in different areas of the brain to aid in the research into neurovascular coupling (Báez‐Yáñez et al., 2020; Havlicek & Uludağ, 2020). Returning to vision, one could apply this method for figure‐ground segregation (Poltoratski et al., 2019; Poltoratski & Tong, 2020; Self et al., 2013) or Kanizsa illusion (Kanizsa, 1976; Kok et al., 2016; Kok & de Lange, 2014) experiments to separate ascending/descending signals across depth by placing the stimulus of interest directly on the location of the pRF in visual space. This drives fMRI from conventional population‐based experimentation to precision‐targeted experimentation, bridging the gap with electrophysiological (rodent/non‐human primate) research.

## 5 CONCLUSION

This work provides a framework for the neuroscientific implementation of line‐scanning fMRI. For neuroscience applications, precise selection and targeting of the cortical location is critical. We propose a method for planning the line based on functional and anatomical properties. Though we selected visual cortical locations, the same framework can select and target other cortical locations. We also discuss how knowledge about the cortical location can be used to design experiments optimal for that cortical location. This strategy is inspired by animal neurophysiology experiments, where extremely high spatiotemporal resolution measurements are performed in a specific part of the cortex. Such an approach is unique for fMRI research and could serve as a guide of what is possible at the extreme end of the spatiotemporal spectrum.

## Supporting information


**Data S1:** Supporting Information.

## Data Availability

The code for this paper is available in the following repositories: Preprocessing of fMRI, anatomical pipeline, and handling of line‐scanning data: linescanning. The described pipeline primarily depends on bash, python, and ANTs. Given the widespread availability of these tools, this pipeline could be implemented at other sites. Successful implementation requires data export to a location where these tools are available. For correct calculation of translation and orientation parameters, one needs to translate the target coordinates back to the coordinate system of the MRI. Line‐scanning experiment: lineprf2. Analysis: pRFline. Data in BIDS‐format (Gorgolewski et al., 2016) will be made available on request.

## Footnotes

^1^
The high resolution is only manifested along the line, that is, 0.25 mm versus 1.7 mm between line‐scanning and whole‐brain acquisitions. This is about a Factor 7. For the overall resolution, however, the line‐scanning resolution is only two times higher; 4 × 2.5 × 0.25 = 2.5 μL for line‐scanning acquisitions, versus 1.7 × 1.7 × 1.7 = 4.9 μL for whole‐brain acquisitions.
