# Supplementary material for: A selection and targeting framework of cortical locations for line‐scanning fMRI
Source: Hum Brain Mapp. 2023 Aug 22;44(16):5471–84. doi: 10.1002/hbm.26459 (PMC10543358; doi:10.1002/hbm.26459)
Supplement: Supplementary file 1 — Data S1: Supporting Information. [file HBM-44-5471-s001.docx]

Supplemental information

1. Supplementary methods

## 1.1. Whole-brain fMRI preprocessing

For each of the BOLD runs found per subject, the following preprocessing was performed as per fMRIprep’s boilerplate (<https://fmriprep.org/en/stable/>): First, a reference volume and its skull-stripped version were generated using a custom methodology of fMRIPrep. A B0-nonuniformity map was estimated based on two (or more) echo-planar imaging (EPI) references with opposing phase-encoding directions, with 3dQwarp (AFNI 20160207). Based on the estimated susceptibility distortion, a corrected EPI (echo-planar imaging) reference was calculated for a more accurate co-registration with the anatomical reference. The BOLD reference was then co-registered to the T1w reference using bbregister (FreeSurfer) which implements boundary-based registration. Co-registration was configured with six degrees of freedom. Head-motion parameters with respect to the BOLD reference (transformation matrices, and six corresponding rotation and translation parameters) are estimated before any spatiotemporal filtering using mcflirt (FSL v5.0.9). BOLD runs were slice-time corrected using 3dTshift (AFNI 20160207). The BOLD time-series were resampled onto the following surfaces (FreeSurfer reconstruction nomenclature): fsnative. The BOLD time-series (including slice-timing correction) were resampled onto their original, native space by applying a single, composite transform to correct for head-motion and susceptibility distortions. These resampled BOLD time-series will be referred to as preprocessed BOLD. Several confounding time-series were calculated based on the preprocessed BOLD: framewise displacement (FD), DVARS and three region-wise global signals. FD was computed using two formulations following Power (absolute sum of relative motions) and Jenkinson (relative root mean square displacement between affines). FD and DVARS are calculated for each functional run, both using their implementations in Nipype. The three global signals are extracted within the CSF, the WM, and the whole-brain masks. Additionally, a set of physiological regressors were extracted to allow for component-based noise correction (CompCor). Principal components are estimated after high-pass filtering the preprocessed BOLD time-series (using a discrete cosine filter with 128s cut-off) for the two CompCor variants: temporal (tCompCor) and anatomical (aCompCor). tCompCor components are then calculated from the top 2% variable voxels within the brain mask. For aCompCor, three probabilistic masks (CSF, WM and combined CSF+WM) are generated in anatomical space. The implementation differs from that of (Behzadi et al., 2007) in that instead of eroding the masks by 2 pixels on BOLD space, the aCompCor masks are subtracted from a mask of pixels that likely contain a volume fraction of GM. This mask is obtained by dilating a GM mask extracted from the FreeSurfer's aseg segmentation, and it ensures components are not extracted from voxels containing a minimal fraction of GM. Finally, these masks are resampled into BOLD space and binarized by thresholding at 0.99 (as in the original implementation). Components are also calculated separately within the WM and CSF masks. For each CompCor decomposition, the k-components with the largest singular values are retained, such that the retained components' time series are sufficient to explain 50 percent of variance across the nuisance mask (CSF, WM, combined, or temporal). The remaining components are dropped from consideration. The head-motion estimates calculated in the correction step were also placed within the corresponding confounds file. The confound time series derived from head motion estimates and global signals were expanded with the inclusion of temporal derivatives and quadratic terms for each. Frames that exceeded a threshold of 0.5 mm FD or 1.5 standardized DVARS were annotated as motion outliers. All resamplings can be performed with a single interpolation step by composing all the pertinent transformations (i.e. head-motion transform matrices, susceptibility distortion correction when available, and co-registrations to anatomical and output spaces). Gridded (volumetric) resamplings were performed using antsApplyTransforms (ANTs), configured with Lanczos interpolation to minimize the smoothing effects of other kernels. Non-gridded (surface) resamplings were performed using mri_vol2surf (FreeSurfer).

# 2. Supplementary Figures


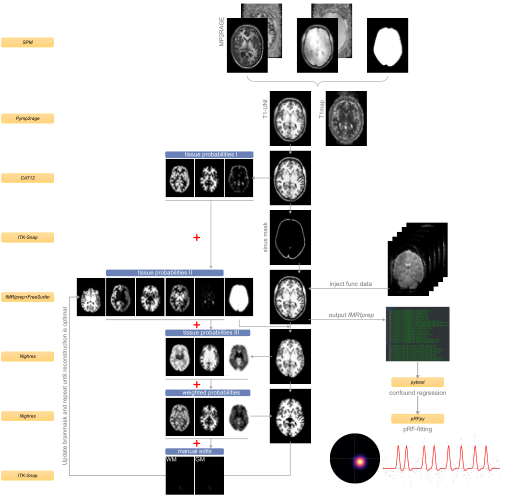


## Figure S1. Overview anatomical and functional preprocessing pipeline. The inversion images from the MP2RAGE scanner were combined to create a unified T1w-image and a T1map. Because this type of sequence induces a noise pattern around the brain which might throw off automated segmentation tools, we used a brain mask from the second inversion image to mask out the noise. From this image, tissue segmentations were obtained with CAT12. A sinus mask was created based on the T1w/T2w-ratio (if T2w-image was present) and manual intervention using ITK-Snap. Voxels within this mask were set to 0 and this final image was injected in fMRIPrep with the --anat-only flag. In parallel, another set of tissue segmentations were obtained from Nighres, which were then combined in a weighted fashion with the segmentations from CAT12 and FreeSurfer. The resulting weighted probability maps were fed into Nighres’ CRUISE algorithm. If needed, gray/white matter were annotated manually, based on which a new brainmask-image was created. The surface of this new image was then reconstructed again as of stage autorecon2. This process was repeated until surfaces were of satisfactory quality. Once finished, we injected the functional data into fMRIPrep, which then used the optimized surfaces to sample the functional data to. The fsnative output from fMRIPrep was then run through pybest, a package that denoises the data based on the confound regressors from fMRIPrep.


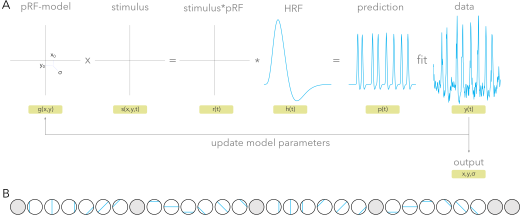


## Figure S2. Population receptive field (pRF) mapping. (A) At every cortical location, the parameters of each model are optimized by minimizing the residual sum of squares (RSS) between the model prediction and the measured BOLD signal. Fitting begins with a grid-search for the best Gaussian model parameters (pRF position and size), and an iterative optimization.


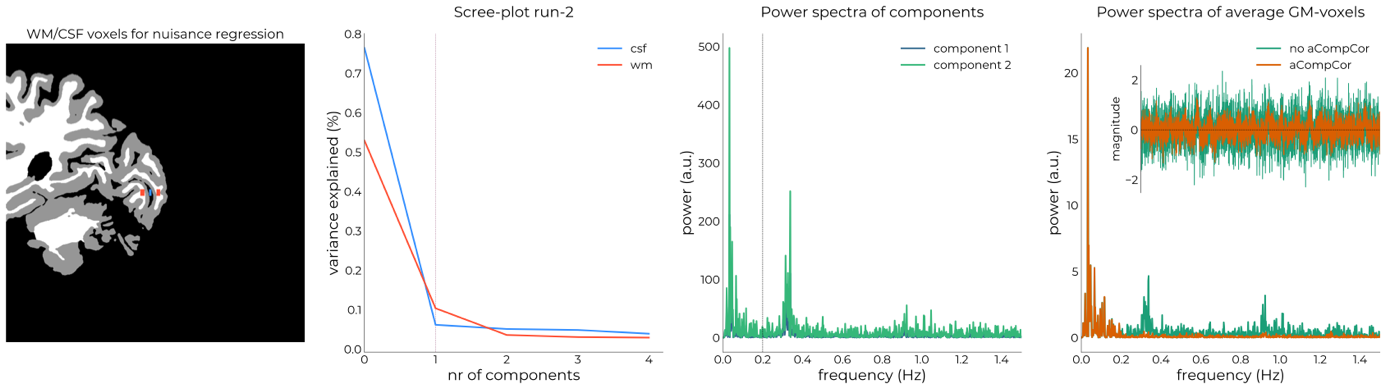


## Figure S3. Example output from the denoising strategy. Nighres segmentation (CRUISE) were projected to the slice, then white matter and CSF voxels along the line were selected (panel 1). Time courses of these voxels were used as inputs for PCA and the resulting components (panel 2) – that show the respiration and cardiac frequencies (panel 3 | respiration = ~0.3Hz, cardiac = ~0.9Hz; Behzadi et al., 2007) were regressed out of the data. Panel 4 showed the frequencies as well as the average gray matter time course before (green) and after (orange) this procedure.


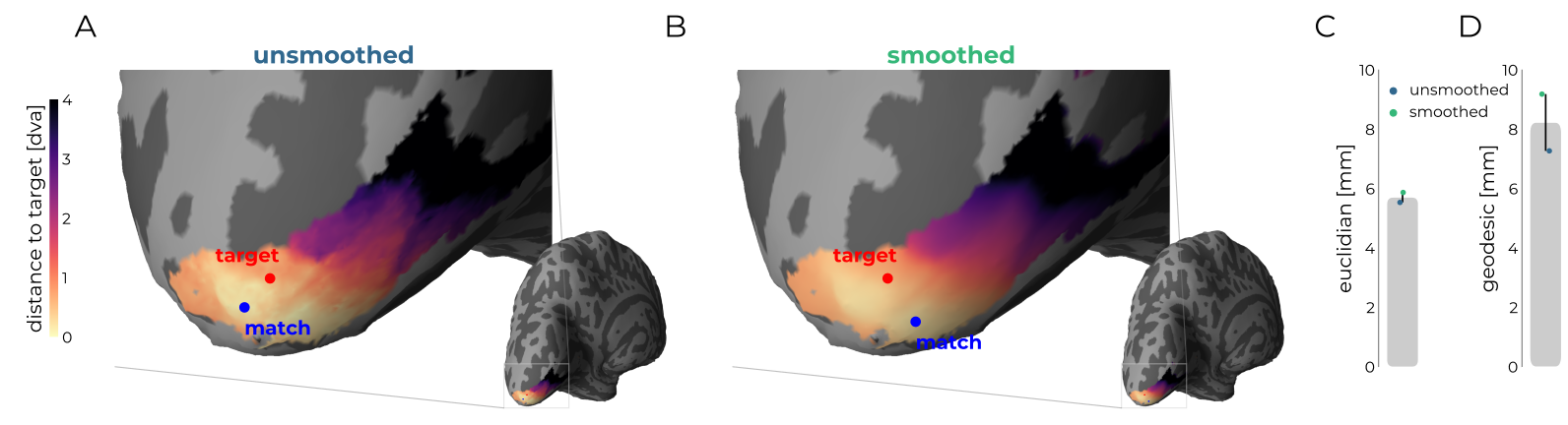


## Figure S4. Smoothing affects distance measures for one subject. (A) An unsmoothed distance map between the vertices in V1 and line-scanning pRF. Without smoothing, the best-matching (blue) pRF lies on one side of the gyrus, whereas after smoothing (B) the best-matching vertex flips to the opposite side. This is not reflected in Euclidean distance (C), but does affect geodesic distance (D) as more surface area needs to be covered.


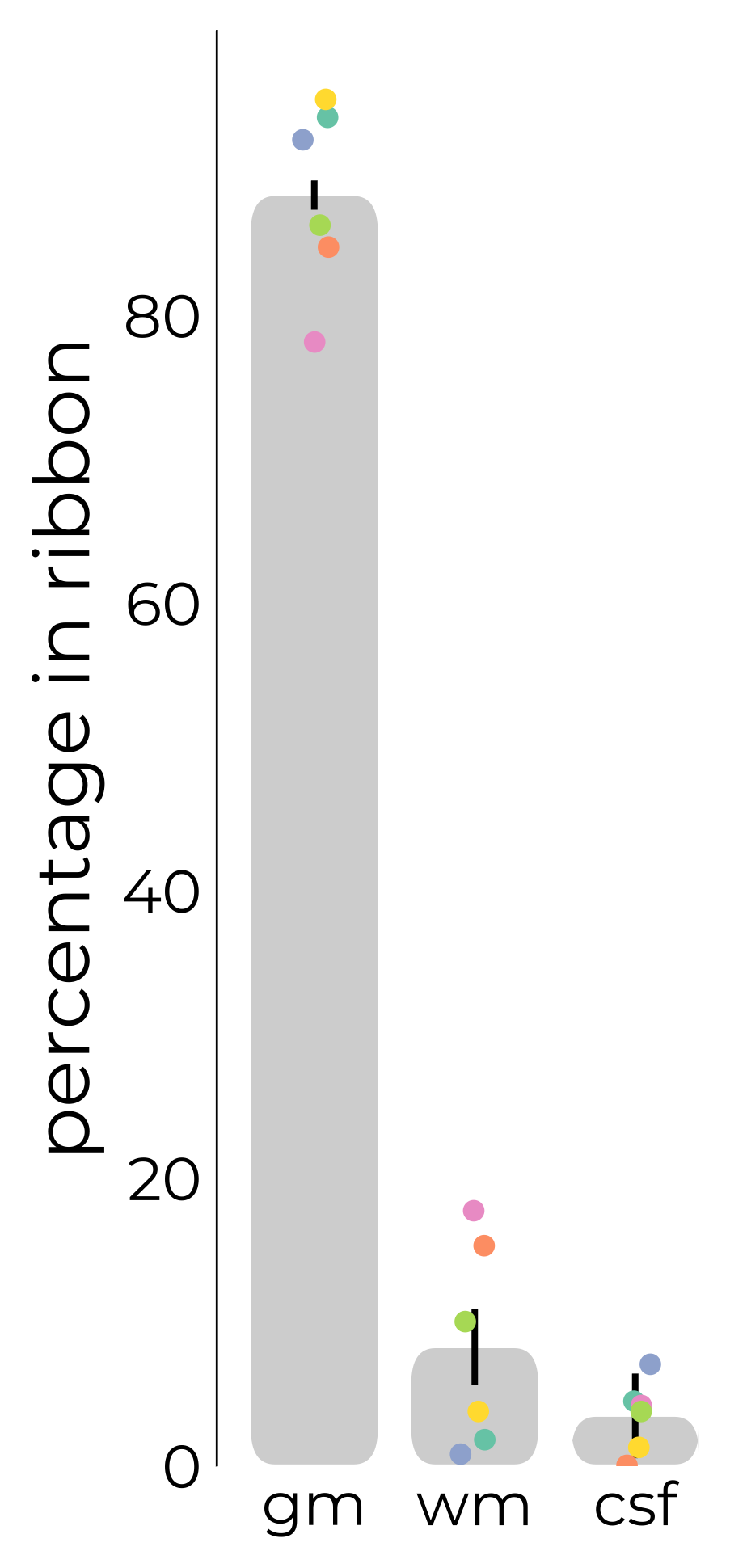


## Figure S5. From manual segmentations, we estimated that the voxels covering the cortical ribbon around the target region consisted of 88.34±1.07% gray matter (GM), 8.22±2.77% white matter (WM), and 3.44±3.18% cerebrospinal fluid (CSF) (Figure S5). These estimations are likely conservative given the imperfect OVS bands. Colored dots indicate individual subjects.

## References

Behzadi, Y., Restom, K., Liau, J., Liu, T.T., 2007. A component based noise correction method (CompCor) for BOLD and perfusion based fMRI. NeuroImage 37, 90–101. https://doi.org/10.1016/j.neuroimage.2007.04.042
